# Minimally Invasive Periodontology: A Treatment Philosophy and Suggested Approach

**DOI:** 10.1155/2021/2810264

**Published:** 2021-06-22

**Authors:** Ethan Ng, John Rong Hao Tay, Marianne Meng Ann Ong

**Affiliations:** ^1^Department of Restorative Dentistry, National Dental Centre Singapore, Singapore 168938, Singapore; ^2^Oral Health Academic Clinical Programme, Duke-NUS Medical School, Singapore 169857, Singapore

## Abstract

Severe periodontitis is a highly prevalent dental disease. With the advent of implant dentistry, teeth are often extracted and replaced. Periodontal surgery, where indicated, could also result in increased trauma to the patient. This literature review discusses different treatment modalities for periodontitis and proposes a treatment approach emphasizing maximum preservation of teeth while minimizing morbidity to the patient. Scientific articles were retrieved from the MEDLINE/PubMed database up to January 2021 to identify appropriate articles that addressed the objectives of this review. This was supplemented with hand searching using reference lists from relevant articles. As tooth prognostication does not have a high predictive value, a more conservative approach in extracting teeth should be abided by. This may involve repeated rounds of nonsurgical periodontal therapy, and adjuncts such as locally delivered statin gels and subantimicrobial-dose doxycycline appear to be effective. Periodontal surgery should not be carried out at an early phase in therapy as improvements in nonsurgical therapy may be observed up to 12 months from initial treatment. Periodontal surgery, where indicated, should also be minimally invasive, with periodontal regeneration being shown to be effective over 20 years of follow-up. Biomarkers provide an opportunity for early detection of disease activity and personalised treatment. Quality of life is proposed as an alternative end point to the traditional biomedical paradigm focused on the disease state and clinical outcomes. In summary, minimally invasive therapy aims to preserve health and function of the natural dentition, thus improving the quality of life for patients with periodontitis.

## 1. Introduction

Periodontitis is a multifactorial and bidirectional inflammatory disease initiated by the accumulation of microbial deposits; nonresolving inflammation and individual susceptibility likewise lead to an overgrowth of periodontal pathobionts which also play a role in the progression of periodontitis [1]. Dysbiosis drives destructive inflammation and perpetuates the disease, and inflammatory byproducts provide nutrients which selectively favour pathobionts and worsen the extent of dysbiosis [2]. These factors codevelop in a reciprocally reinforced way, leading to a disease state in the susceptible individual [3]. Since tissue damage may be attributed to the host inflammatory response, cytokines represent an intermediate mechanism between bacteria and host-mediated tissue destruction [4]. The use of salivary cytokines as biomarkers of periodontitis has utility in personalised dentistry, especially for the identification of higher-risk patients or nonresponders and to determine disease activity and progression [5].

**Figure 1:**
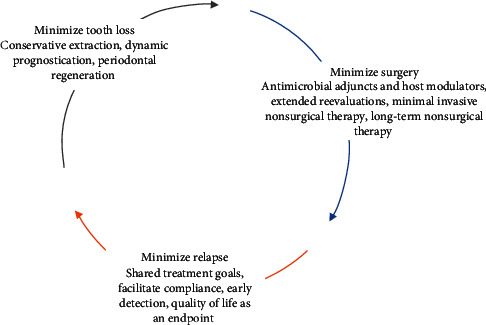
The minimally invasive approach to periodontology involves three interlinked goals. This aims to minimize tooth loss at the initial stage and minimize surgery by considering the use of adjuncts and appropriate evaluation periods. If indicated, regenerative surgery with a minimally invasive approach may improve attachment levels. Minimizing relapse will in turn minimize tooth loss and improve the quality of life.

With a prevalence of 35% and 11.2%, respectively, untreated dental caries and severe periodontitis are two of the most common dental diseases [6, 7]. While their aetiologies may differ, both conditions involve establishing a dysbiotic polymicrobial community influenced by host factors and inflammation, whose activities converge to initiate the disease [8]. Both conditions may also result in tooth loss, which is associated with an impaired oral health-related quality of life [9]. Thus, the prevention and control of caries and periodontitis should be an aim of every dentist. The concept of minimally invasive dentistry is based on tissue preservation; preferably by prevention and early intervention and then by minimizing tissue loss should intervention be required [10]? It is an evolving treatment philosophy in healthcare based on scientific advances and a more recent focus on patient-reported outcome measures. In cariology, this has taken the form of early diagnosis, remineralization of early lesions, and minimally invasive cavity preparation techniques [11]. In periodontology, this concept has focused mainly on surgical techniques that minimize surgical trauma, thus optimizing wound healing and reducing patient morbidity [12]. Therefore, this literature review aims to discuss periodontitis and different treatment modalities, with the purpose of integrating these into a holistic treatment approach which includes a patient's quality of life as an endpoint (Figure 1). The effectiveness of this approach is discussed in terms of clinical performance and patient perception.

## 2. Materials and Methods

A literature search was conducted in MEDLINE/PubMed up to January 2021 to identify appropriate articles for this review. A variety of MeSH terms and keywords were employed in keyword/title/abstract searches, including minimally; invasive; minimally invasive surgical procedures; minimally invasive non-surgical procedures; periodontics; periodontology; periodontal disease; prognostication; tooth extraction; tooth loss; periodontal regeneration; non-surgical, adjunctive therapy; host modulators; anti-infective agents; biomarkers; periodontal reviews; and periodontal maintenance. The definition for a minimally invasive procedure was “any intervention described as minimally invasive.” Articles were screened by title, abstract, and full text for relevance. Full-text examination was also carried out for studies with insufficient information from the titles or abstracts to make a definitive decision. Related articles published in English, reviews, meta-analyses, and clinical studies in humans were included. Case reports, implant-associated surgeries (e.g., sinus lift surgery, socket preservation, or bone grafting), soft tissue or other restorative dentistry procedures, and animal and *in vitro* studies were excluded. The electronic search was supplemented by cross-checking the bibliographies from identified articles.

## 3. Minimizing Tooth Loss

Periodontitis is a major cause of tooth loss among adults; the downstream sequelae include loss of self-esteem, quality of life, and masticatory dysfunction which may compromise nutrition and general health [13]. The 2017 World Workshop classification considers the number of teeth lost due to periodontitis as a stage defining the level of severity, with ≥5 teeth possibly leading to the need for complex rehabilitation [14]. Inherent in the process of tooth loss is the assignment of tooth prognosis, which has traditionally been interpreted as the probability of tooth mortality.

### 3.1. Predictive Value of Tooth Prognostication

Various classification systems exist, but it is unclear if any system is superior to the other. What is clear, however, is that tooth prognosis is influenced not only by clinical and radiographic findings but also by the control of modifiable behavioural risk factors and quality of received treatment [15, 16]. Furthermore, the clinician that assigns the prognosis and performs the corresponding treatment dictates the outcome of the tooth [17]. In a study of Norwegian dentists, a low forceps level (threshold for extraction) resulted in the removal of 60% teeth with ≤50% periodontal support [18]. Some clinical guidelines have proposed that teeth with bone loss >65% have a poor long-term survival, adding to the decision for extraction [19, 20]. However, the assignment of prognosis is complicated by a wide diversity of different possible cases which a tooth prognostication system may not adequately address. The decision to extract teeth should also not be based on mobility alone. Since the predictive power of a tooth with a prognosis other than “good” is approximately 50% [21], a more conservative approach in extracting teeth ought to be followed. In support of this, long-term studies have demonstrated the retention of teeth with even a questionable prognosis and advanced furcation involvement [22–24].

### 3.2. Periodontal Regeneration

Periodontal regeneration may be defined as “the restoration of lost or diminished periodontal tissues, including the cementum, periodontal ligament, and alveolar bone [25]. If successful, it may increase periodontal support and remains the only modality that may result in regression of a periodontal stage [26]. Recently, a systematic review reported that periodontal regeneration of intrabony defects resulted in improved attachment levels and a higher rate of tooth survival over a period of up to 20 years [27]. Furthermore, the treatment of intrabony defects with guided tissue regeneration or with biomaterials such as enamel matrix derivative performs better than open flap debridement [28], and these results are maintained in the long term, even in teeth with furcation involvement [29, 30]. Minimally invasive approaches to periodontal regeneration have also shown to be effective over the long term and will be discussed later [31, 32]. Thus, periodontal regeneration plays an important role in improving the prognosis of a tooth and should be attempted with a minimally invasive surgical approach when indicated.

## 4. Minimizing the Need for Surgery

The presence of residual pockets ≥5 mm and a full-mouth bleeding score >30% after nonsurgical therapy represents a risk factor for disease progression and tooth loss and therefore may be regarded as an incomplete periodontal treatment outcome [33–35]. Persistent pockets are commonly perceived as requiring “additional therapy,” usually following a surgical course. A major advantage of surgical therapy is visual access, and clinicians often fail to completely debride roots of plaque and calculus, especially in the presence of deep pocketing [36].

An important prerequisite prior to any periodontal surgery is the optimization of patient plaque control. Successful outcomes are obtained in patients with good plaque control, whereas periodontal surgery in plaque-infected dentitions results in disease recurrence and significant loss of further attachment [37, 38]. In addition, the maintenance of a high standard of oral hygiene is strongly associated with a good regenerative outcome [39, 40]. The use of appropriate interdental cleaning aids, antimicrobial adjuncts, and sessions of repeated instrumentation with oral hygiene reinforcement may also possibly result in further probing pocket depth reductions in the nonsurgical phase [41], obviating the need for surgical therapy.

### 4.1. Use of Antimicrobial Adjuncts and Host Modulators

Periodontitis follows a polymicrobial model of community dysbiosis, and the transition to disease resembles ecological succession that culminates in a higher proportion of oral pathobionts [42]. As nonresolving inflammation is thought to drive this conversion, the application of host modulation, which refers to manipulation of the immune response to prevent or ameliorate tissue damage, may facilitate an environment that reverses dysbiosis and promotes repair [2]. These topics were the subject of systematic reviews covered in the XVI European Workshop in Periodontology. Adjunctive antiseptics were found to significantly reduce inflammation, and locally delivered or systemic antibiotics were associated with significant clinical benefits [43–45]. Of note were the more frequent incidence of adverse events and growing concerns with antibiotic resistance with the use of systemic antimicrobials, calling into urgent action the need to look for alternatives in the long term. The combination of mechanical debridement with host modulators may improve treatment outcomes, and locally delivered statin gels and subantimicrobial-dose doxycycline have been identified as potentially effective modalities [46]. Although the efficacy of probiotic therapy is equivocal and current evidence does not support routine use, future adequately powered multicentre trials may further elucidate its promising role in minimally invasive periodontology [46–48].

### 4.2. Extended Reevaluations or Surgery?

The efficacy of single or repeated rounds of debridement in improving periodontal status has been debated in the literature. In a study involving severely advanced periodontitis patients, Badersten et al. observed that a single episode of instrumentation was effective in treating deep periodontal pockets, compared to repeated instrumentation performed three times at intervals of two to four months [49]. Similarly, another study by Anderson et al. stated that multiple episodes of instrumentation are ineffective, although in this study, the interval for additional instrumentation was 24 hours apart [50]. The authors concluded that if calculus was not removed after one episode of debridement, repeated instrumentation was unlikely to change that. However, more recent evidence from several other studies supports clinically significant further reductions after the initial debridement, and they were more pronounced for moderate and deep pockets [51–53].

Thus, it should be noted that the results of classic studies such as Badersten et al. and Anderson et al. may not be generalized. Indeed, a single episode of scaling often does not result in complete removal of subgingival calculus, with deeper pockets associated with less complete calculus removal [54]. The quality of instrumentation is also dependent on the clinician skill and time allocated for the procedure. Hence, treated areas should be reevaluated at an appropriate time after initial inflammation subsides, and reinstrumentation was performed before considering periodontal surgery. Notably, it takes time for the periodontium to remodel after a single round of nonsurgical periodontal therapy. The traditional reevaluation of therapy at 8–12 weeks may mask the real potential of nonsurgical treatment as healing and maturation of the periodontium occur over the next 9–12 months [55–57]. Waiting for an extended period before surgery is also recommended to assess the patient's compliance to recall appointments and plaque control before any surgical intervention is carried out [58].

### 4.3. Minimally Invasive Nonsurgical Therapy (MINST)

A minimally invasive approach to nonsurgical therapy was first proposed by Ribeiro et al. Root surface debridement was achieved with slim ultrasonic tips and minicurettes, and care was taken to preserve the stability of soft tissues [59]. Using clinical and radiographic evidence, this approach demonstrated similar efficacy to the minimally invasive surgical technique in the treatment of intrabony defects. In another study, a mean pocket closure of 71.6 ± 15.7% was observed for sites with an initial probing depth of >5 mm at the patient level, indicating a similar if not better success rate compared to traditional nonsurgical approaches [60]. Nibali et al. assessed the healing of defects with a radiographic intrabony component >3 mm following MINST in nonsmokers, and significant clinical and radiographic improvementswere similarly observed [61]. These improvements were found to be stable after a five-year reassessment [32]. Aimetti et al. evaluated the addition of the enamel matrix derivative to ≥3 mm intrabony defects following a MINST or MIST (minimally invasive surgical technique) approach, and the MINST approach was found to have comparable clinical outcomes and a shorter treatment time [62].

Healing following nonsurgical therapy typically begins with the formation of a blood clot, with eventual formation of long junctional epithelium. It is speculated that the MINST approach results in a more stable blood clot in the intrabony defect, which may result in bone apposition or an increase in bone mineralization after nonsurgical therapy [61]. Similar to the minimally invasive approach to surgery, preservation of supracrestal periodontal fibre attachment to the cementum is also an important requirement [63]. These observations indicate the potential of nonsurgical therapy as a valid treatment option for intrabony defects. However, more clinical evidence is required, and a clinical trial comparing a minimally invasive surgical and nonsurgical approach is underway [64].

The periodontal videoscope, introduced in 2002, is also worth a mention. By enabling direct real-time visualization and magnification of the subgingival tooth surface, improvement in the quality of nonsurgical debridement and healing outcomes could be achieved [65–67]. However, the considerable cost of the device, lack of studies demonstrating its efficacy, steep learning curve, and resistance to change from conventional techniques have meant that this device is still not widely used [68].

### 4.4. Long-Term Studies on the Comparative Efficacy of Surgical Access Flaps and Nonsurgical Approaches

A recent systematic review on the efficacy of access flap surgery compared to nonsurgical debridement demonstrated that greater probing depth reductions are achieved in deep pockets undergoing surgical therapy compared to nonsurgical therapy, but these differences reduce over time with an additional effect of 0.5 mm in the long term [69]. Moderately deep pockets (4–6 mm) had no differences in the outcome regardless of whether surgery was done. In a network analysis comparing minimally invasive nonsurgical and surgical techniques, nonsurgical techniques had a lower probability to be the best treatment option in terms of probing depth reduction and clinical attachment gain, but limited studies and heterogenous data preclude a conclusive statement on this matter [70]. Thus, both nonsurgical and surgical treatment options may be considered effective in the long term as a reduction in probing depth after surgery may not be sustained. It is important to mention that many of the classic longitudinal studies only employed the use of access flaps such as the modified Widman flap, and these findings may not be applied to regenerative approaches. However, this observation also suggests that factors other than the treatment performed are important in sustaining pocket reduction, such as control of modifiable risk factors and patient compliance to a maintenance program.

## 5. Minimizing Relapse

Periodontology is one of the few medical and dental disciplines with the privilege of having long-term data, and the 30-year maintenance results of Axelsson et al. demonstrate conclusively that a maintenance program tailored to individual needs may result in near-complete periodontal stability [71]. A more recent study by Graetz et al. of up to 18 years demonstrated that long-term tooth retention is possible with a structured maintenance program, regardless of the patient baseline characteristics [72]. Thus, potential intervention (surgery or extraction) due to longitudinal deterioration of the periodontium may be minimized by facilitating patient compliance, early detection using biomarkers, and considering the quality of life as an endpoint of therapy in tandem with clinical measures.

### 5.1. Shared Treatment Goals and Facilitating Compliance

Using a definition of patients who missed <30% of scheduled maintenance visits or who never went two years without a maintenance visit, the number of complete compliers over 20 years of observation was one in three [73, 74]. This is significant, and it is well documented in the literature that erratic compliers have more tooth loss and alveolar bone loss, resulting in a greater impact on the daily performance (estimated by the functional performance, emotional stability, and social performance) [74–76]. Furthermore, the effect of poor prognosis teeth having a detrimental effect on adjacent teeth increases with less regular recall intervals [77, 78]. These observations underscore the importance of the role of both clinician and patient in working towards a shared treatment goal as both stakeholders may have initially different perceptions and endpoints in resolving the pathology of the disease. Patient factors influencing compliance are not well substantiated in the literature [79]. Noncompliance is the net result of complex behavioural patterns and factors such as stressful life events, low self-efficacy, initial presentation of acute symptoms, and lack of copayment options [80–82]. The lack of motivation appears to be an important factor in determining compliance [79], which highlights the importance of patient education and behavioural modification strategies such as goal setting, self-monitoring, and motivation interviewing [83, 84]. Indeed, personalised oral health education has low additional incremental costs and has been found to be both more clinical and more cost-effective than standardized oral health education programs [85–87]. Finally, the use of air-polishing devices for periodontal maintenance is associated with a more favourable patient perception and may have utility in improving compliance [88].

### 5.2. Utilising Biomarkers for Early Detection of Periodontitis

The use of biomarkers is emerging as an important tool for monitoring periodontal health status. The inflammatory process of periodontitis is mediated by complex cytokine interactions at the cellular and molecular level, and they exert regulatory control over immune cells in the periodontium [4, 89]. Furthermore, virulence factors produced by periodontal pathobionts may mediate the immune response in favour of inflammation [90, 91]. Potentially diagnostic biomarkers are related to inflammation (interleukin-1-beta, interleukin-6, and tumour necrosis factor-alpha), connective tissue degradation (matrix metalloproteinase (MMP)-8 and -9, tissue inhibitors of metalloproteinase (TIMP)-1, and aspartate aminotransferase), and alveolar bone resorption (osteoprotegerin and carboxy-terminal telopeptide of type 1 collagen), as well as growth factors such as hepatocyte growth factor [92–94]. Indeed, the balance of cytokine profile may determine progression and severity of the disease [95, 96].

Biomarkers may be derived from gingival crevicular fluid (GCF), which carries site-specific immunological content [97], or saliva, which is a pooled surrogate fluid to GCF that provides information on the overall state of the oral environment [98]. Serum biomarkers, on the contrary, may not play an important diagnostic or predictive role as plasma cytokine levels could be unrelated to the inflammatory changes occurring in periodontal tissues [99, 100]. In another study, the greatest sensitivity came from saliva biomarkers, and the greatest specificity was observed for GCF biomarkers [101]. More recently, however, newer biomarkers such as galectin-3 and nod-like receptor family pyrin domain-containing protein-3 (NLRP3) inflammasome released during inflammatory conditions in the serum and saliva may discriminate between a state of periodontitis and periodontal health [102, 103].

The use of biomarkers is especially relevant in point-of-care treatment, where saliva or gingival crevicular fluid can be collected noninvasively from the patient, the former potentially being used for patient-specific screening while the latter being used for site-specific identification of disease activity [97]. Indeed, the reliability of GCF in identifying cytokines is comparable to more invasive techniques such as gingival biopsies and blood serum, the latter being found to be the least sensitive [104]. It is likely necessary to employ holistic approaches in identifying multiple biomarkers in specific combinations for this purpose, rather than single or limited numbers of biomarkers [105, 106]. For example, classification and regression tree analysis of a panel of salivary biomarkers including MMP-9, TIMP-1, and IL-1b was able to predict patients with periodontitis with an accuracy of close to 100% [106]. The advent of high-throughput gene microarray analysis and large-scale proteomics may greatly contribute to the prediction and investigation of sites with disease activity, though this aspect remains largely unexplored [97, 98], in part due to issues such as high costs and low recoverability of low-abundance cytokines [107].

### 5.3. Quality of Life as an Endpoint in Periodontal Therapy?

Minimally invasive periodontology should also comprise a holistic approach to patient care, which does not only focus on a biomedical paradigm that focuses on disease states and clinical outcomes but also on the psychological and social aspects related to the oral cavity. Tools that have been used to measure the quality of life in the context of the oral cavity include the General Oral Health Assessment Index (GOHAI) [108], the Dental Impact Profile [109], and the Oral Health Impact Profile [110, 111]. While subjective health measures are often faced with complexities in measuring change, it can be combined with objective assessments and is potentially useful in predicting treatment need [112].

From a patient perspective, both surgical and nonsurgical periodontal therapy significantly influence oral health-related quality of life scores [113, 114]. Nonsurgical treatment has also been shown to effectively reduce psychological discomfort and dental pain over the long term [115]. Interestingly, no further improvement to oral health-related quality of life scores was reported after surgical therapy, indicating that any added clinical benefits and necessity for the procedure may not be perceived by patients. On the contrary, no difference in postsurgical pain and discomfort could be detected by patients when comparing minimally invasive nonsurgical and minimally invasive surgical approaches, suggesting that minimally invasive surgical approaches may be the preferred option if possible [70]. These results come with a caveat as the silent and chronic course of periodontitis progression means that the current instruments for evaluating the quality of life in patients with periodontitis before and after periodontal therapy may not be adequately sensitive enough to detect changes, and though there have been calls to take quality of life factors into consideration in assessing treatment needs and approaches in the periodontal patient, clinical decisions should still be assessed together with objective clinical outcome measures [35, 116].

## 6. Minimally Invasive Surgical Therapy

Minimally invasive therapy should first focus on improving the prognosis of the remaining dentition through nonsurgical therapy and adjunctive therapy where applicable and only progress to surgical therapy if this approach fails. The minimally invasive approach to surgery has been described as early as 1998, where Harrel described a surgical technique utilising small incisions and minimal flap reflection for the treatment of periodontal defects [117]. Clinical results for this technique were similar to traditional surgical approaches involving larger flaps, with added benefits of reduced postoperative pain, improved rate of healing, maintenance of soft tissue height, and patient acceptance [118, 119]. Minimally invasive surgery subsequently evolved into a microsurgical approach, described as the “minimally invasive surgical technique” by Cortellini and Tonetti [120, 121]. The microsurgical approach facilitated gentle handling of tissues and precise wound closure, resulting in *a* > 90% rate of primary closure reported by some studies [120–122]. Other advantages include improved visual acuity, enhanced revascularization, and maximum preservation of the tissue [123, 124]. Various double-layer suturing approaches have been suggested when a microsurgical approach is used [120, 125, 126]. Today, key aspects of a “minimally invasive” surgery are as follows: use of magnification, minimal flap extension and reflection, preservation of the papilla and interproximal supracrestal soft tissue, and precise suturing technique. Various designs for a minimally invasive surgical approach have been developed, with smaller flaps designed to optimize wound stability, flap integrity, and healing by primary intention (Table 1).

Subsequent systematic reviews and meta-analyses have established that regenerative outcomes with a minimally invasive surgery are superior to access flaps [28, 124]. Furthermore, the additional use of biomaterials or membranes has been used in combination with a minimally invasive surgery with successful outcomes of up to six years and low postsurgical morbidity [31, 132–137] but does not appear to enhance regenerative outcomes, indicating enhanced healing potential with a minimally invasive surgical approach [138–141]. Even if a minimally invasive approach is adopted, factors such as patient compliance to professional recalls and plaque control and inherent anatomical risk factors such as defect configuration and gingival phenotype may influence successful outcomes [58, 142]. At least two studies have compared a minimally invasive double-flap to the minimally invasive single-flap approach, and a better quality of early wound healing and lower postoperative pain were observed in the latter [143, 144].

## 7. Discussion

This literature review discussed a proposed treatment approach emphasizing maximum preservation of teeth while minimizing morbidity to the patient. Comparison of minimally invasive surgical and nonsurgical procedures published in the literature showed that associated clinical results are not inferior to traditional approaches. Additional benefits have been identified, such as increased clot stability, improved healing, enhanced revascularization, higher incidence of primary closure, and better patient acceptance. Minimally invasive periodontology is often discussed in the context of clinical procedures, as shown by the literature search. However, the term “minimally invasive” should encompass a holistic philosophy that includes treatment planning from the first appointment. With the advent of implant dentistry, tooth extractions have been indicated much more often. Dogmas that have been propagated include (1) “periodontal overtreatment” or the claim that continued periodontal treatment in an attempt to maintain periodontally involved teeth compromises the surgical site due to continued bone loss; (2) full clearance of remaining periodontally compromised teeth reduces the risk of reinfection [145]. On the contrary, we argue that a patient's perception of the usefulness of the tooth, i.e., functional, not unaesthetic, and no physical, psychological, or social discomfort, may be used as a valid justification to retain and maintain a tooth nonsurgically, even if it may be considered by the clinician to be of a poor prognosis. Implicit in the decision to retain such teeth would be the joint understanding that compliance to a structured maintenance program is necessary. Indeed, the long-term longevity of implants does not exceed healthy teeth or even teeth that are compromised but adequately treated and maintained [146–148]. Implants are not supposed to replace teeth; they are only meant to replace missing teeth [149]. The association of biomarkers of inflammation in saliva or GCF with periodontitis also provides a noninvasive way for early detection of periodontitis or estimation of disease activity and risk. As significant damage is required before clinical signs of periodontitis appear, this has implications in timely management or early intervention, which is consistent with a minimally invasive approach. Rapid chairside testing also facilitates personalised treatment, where specific sites can be targeted for further intervention while minimizing postinstrumentation morbidity to stable sites.

## 8. Conclusions

A minimally invasive approach is associated with noninferior outcomes to traditional approaches. Added advantages include enhanced healing potential, recession reduction, improved patient acceptance, and personalised and targeted treatment. While many studies have published outcomes of periodontal therapy in terms of probing depths and clinical attachment gain, there are other units of measurement that must be considered when evaluating the effectiveness of therapy, such as continued dental functionality and patient-reported outcomes. The broader end goal of periodontal therapy should lie in improving the quality of life for patients, which involves prioritizing the health, function, and long-term success of the natural dentition. Thus, we propose a holistic approach to managing periodontal patients in a minimally invasive manner.

## Figures and Tables

**Table 1:** Various designs for a minimally invasive surgery to facilitate wound stability and improved regenerative outcomes.

Study	Technique	Key design features	Remarks
Harrel [119]	Minimally invasive surgery (MIS)	Two separate intrasulcular incisions with a connecting single horizontal incision (at the palatal aspect for aesthetic areas) placed 2-3 mm from the papilla crest.	Performed with at least 3.5x magnification, a granulation tissue-removing instrument, and high-speed finishing bur for root surface smoothening.

Cortellini and Tonetti [120]	Minimally invasive surgical technique (MIST)	Mesiodistal extensions kept to a minimum, avoid vertical releasing incisions, and elevate only the defect-associated papilla where possible.	Modified papilla preservation flap or simplified papilla preservation flap for the interdental incision, extended to buccal and lingual aspects. A microsurgical approach with ×4–16 magnification and enamel matrix derivative are used.

Cortellini and Tonetti [121]	Modified minimally invasive surgical technique (M-MIST)	Mesiodistal extension extends only to the midbuccal area of involved teeth.	Modified papilla preservation flap or simplified papilla preservation flap for the interdental incision, only extended to the buccal aspect. A microsurgical approach with ×4–16 magnification and enamel matrix derivative are used.

Trombelli et al. [127]	Single-flap approach (SFA)	Only an envelope flap on the buccal and oblique or horizontal incisions interproximally following the profile of the underlying bone crest. Interproximal supracrestal gingival tissues are left intact.	×2.5 magnifying loupes are used. Limited to intraosseous defects requiring buccal access. May be more suitable with an enamel matrix derivative ± bone graft approach, rather than membrane + bone substitute due to wound dehiscence [128, 129].

Aslan et al. [130]	Entire papilla preservation flap (EPP)	Buccal intracrevicular and single short vertical releasing incision, followed by interdental tunnel preparation below the papilla to access the defect.	Microsurgical instruments, surgical loupes ×3.3, and a specifically designed angled tunnel elevator are required. Regenerative material consisted of enamel matrix derivative + bone substitute.

Moreno Rodriguez and Caffesse [131]	Nonincised papilla surgical approach (NIPSA)	Buccal horizontal incision apical to the periodontal defect, followed by raising the flap coronally, allowing surgical access to the defect without disrupting marginal tissues.	Root surface debridement performed up to the first 2-3 mm of the pocket in question during the nonsurgical phase to preserve fibers attached to the root and to prevent postoperative shrinkage. ×2.8 magnifying loupes are used, and regenerative material consisted of enamel matrix derivative + bone substitute.

## Data Availability

The data used to support the findings of this study are included within the article.
